# Critical Care Management Considerations in *Klebsiella pneumoniae* Invasive Liver Abscess Syndrome: Case Reports and Narrative Review

**DOI:** 10.1155/crcc/5579095

**Published:** 2026-06-22

**Authors:** Jerry Chen, Caleigh Horan, Krishen Wadhwani, Pedro M. Dammert, Elizabeth Sophy

**Affiliations:** ^1^ Department of Internal Medicine, San Ysidro Health, San Ysidro, California, USA; ^2^ Department of Internal Medicine, Scripps Mercy Hospital, Chula Vista, California, USA; ^3^ Department of Pulmonary and Critical Care, Scripps Mercy Hospital, Chula Vista, California, USA; ^4^ Department of Palliative Care Medicine, Scripps Mercy Hospital, Chula Vista, California, USA

**Keywords:** critical illness, endophthalmitis, *Klebsiella pneumoniae*, liver abscess, meningitis

## Abstract

**Importance:**

*Klebsiella pneumoniae* invasive liver abscess syndrome (ILAS) is an emerging disease characterized by liver abscess without biliary disease, often with multiorgan metastatic infection. It can cause severe critical illness with significant morbidity and disability. There remains limited literature and understanding of this syndrome in the United States.

**Objectives:**

This article aims to describe the clinical characteristics of hospitalized and critically ill patients with ILAS and providing management considerations for critical care clinicians.

**Main Outcomes and Measures:**

Adult patients admitted to Scripps Health from Janurary 1, 2018 to April 1, 2024 were reviewed. Inclusion criteria required radiographic evidence of a liver abscess and cultures (blood or abscess) positive for *K.* *pneumoniae*. Data collected included demographics, symptoms and signs, laboratory and radiologic data, metastatic infection characteristics, treatment, mortality, and morbidity. Patient cases were described, and a narrative review describing clinical, therapeutic, and prognostic characteristics was conducted.

**Results:**

Six patients had ILAS. Mean age was 54.8 years, no immunosuppression (defined as underlying malignancy, drug‐induced, or chronic infection), 83% had diabetes, 50% were male, and 50% were of Asian ethnicity. Liver abscesses were multilocular (50%), and all had percutaneous catheter drainage. Fifty percent of patients developed distant metastatic infections: emphysematous cystitis, pulmonary septic emboli, complicated parapneumonic pleural effusions, meningitis, ventriculitis, cerebral septic emboli, and endophthalmitis. These patients were younger, male, and had a higher rate of intensive care unit (ICU) admission and mechanical ventilation. Two patients required ICU admission with septic shock, diabetic ketoacidosis, respiratory failure, severe thrombocytopenia, and altered mental status. *K*. *pneumoniae* isolates were pansensitive except to ampicillin. There was no in‐hospital mortality. The patient with meningitis and ventriculitis had full neurological recovery, and the patient with endophthalmitis had very poor residual visual acuity.

**Conclusions and Relevance:**

ILAS is an emerging disease that can cause severe critical illness with multiorgan involvement. Early identification of the disease and metastatic infection is essential to provide appropriate treatment. Additionally, ILAS patients require screening for endophthalmitis.

## 1. Introduction

*Klebsiella pneumoniae* invasive liver abscess syndrome (ILAS) is a distinct, community‐acquired syndrome of liver abscess without hepatobiliary disease. Patients are usually diabetic, immunocompetent, middle‐aged, and many develop metastatic infection, predominantly pulmonary septic emboli, endophthalmitis, meningitis, and brain abscesses [1, 2]. Hypervirulent *K*. *pneumoniae* strains with hypermucoviscous phenotype, particularly serotypes K1 and K2, are associated with ILAS. Hypervirulent *Klebsiella* has been increasingly identified and associated with more severe pathogenicity with distant metastatic infection. This is contrasted with the classical strain that has less hypermucoviscosity. Various virulent factors have been identified, including different capsule serotypes, siderospheres, lipopolysaccharide, and fimbriae [3–5].

Initially described in the 1980s in Taiwan [6], ILAS is now a globally emerging disease increasingly reported in North America. There exists an Asian ethnicity predominance among cases, but it also occurs in non‐Asian individuals without recent travel to endemic areas [7].

As many of these patients are critically ill and require high clinical suspicion for a correct and timely diagnosis, our goal is reporting our experience with hospitalized and critically ill patients with ILAS and to provide management considerations for critical care clinicians.

## 2. Methods/Materials

These case reports were conducted on patients admitted to Scripps Hospital Systems in Southern California from January 1, 2018 to April 1, 2024. Inclusion criteria were adult hospitalized patients with liver abscess on imaging and either abscess or blood cultures positive for *K*. *pneumoniae*. After IRB approval, patients were identified from Epic EMR Slicer Dicer using ICD codes for liver abscess and culture results positive for *K*. *pneumoniae* in blood or liver abscess aspirate. Manual extraction was utilized to collect data variables, including demographics, clinical characteristics, laboratory values, radiographic findings, treatment course, and clinical outcomes. Data were synthesized with descriptive analysis.

## 3. Case Reports

Nine patients met inclusion criteria. Six had no underlying hepatobiliary disease (ILAS) and three had underlying hepatobiliary disease (secondary liver abscess).

### 3.1. Invasive Liver Abscess Syndrome

Patients had a mean age of 54.8 years, no immunosuppression (defined by underlying malignancy, drug‐induced, or chronic infection), and 83.3% had diabetes. Fifty percent were male, 50% were of Asian origin, 16% had a recent trip to the Philippines, and 33.3% required ICU admission and mechanical ventilation (Table 1).

**Table 1:** Demographic and clinical data, treatment, and outcomes of patients with ILAS.

Variable	All ILAS (six patients)	ILAS without metastatic infection (three patients)	ILAS with metastatic infection (three patients)
Age (mean)	54.8	68.6	41
Male	50% (3)	0	100% (3)
Asian origin	50% (3)	66.6% (2)	33.3% (1)
Immunosuppression	0	0	0
Malignancy	0	0	0
Diabetes	83.3% (5)	66.6% (2)	100% (3)
Fever	66.6% (4)	100% (3)	33.3% (1)
Abdominal pain	50% (3)	100% (3)	0
Nausea and vomiting	50% (3)	100% (3)	0
Shortness of breath	33.3% (2)	33.3% (1)	33.3% (1)
Altered mental status	33.3% (2)	0	66.6% (2)
Headache	33.3% (2)	33.3% (1)	33.3% (1)
Leukocytosis	50% (3)	66.6% (2)	33.3% (1)
Elevated LFTs	83.3% (5)	66.6% (2)	100% (3)
Elevated bilirubin (> 1.3 mg/dL)	50% (3)	33.3% (1)	66.6% (2)
Mean bilirubin	1.6	1.53	1.8
Coagulopathy (INR > 1.5)	0	0	0
Septic shock	33.3% (2)	0	66.6% (2)
DKA	33.3% (2)	0	66.6% (2)
A fib RVR	16.6% (1)	0	33.3% (1)
ICU admission	33.3% (2)	0	66.6% (2)
Mechanical ventilation	33.3% (2)	0	66.6% (2)
Severe thrombocytopenia	33.3% (2)	0	66.6% (2)
Acute renal failure	33.3% (2)	0	66.6% (2)
Pulmonary septic emboli	50% (3)	0	100% (3)
Complicated parapneumonic effusions	33.3% (2)	0	66.6% (2)
Urinary tract infection	33.3% (2)	0	66.6% (2)
Cerebral septic emboli	33. % (2)	0	66.6% (2)
Meningitis	16.6% (1)	0	33.3% (1)
Ventriculitis	16.6% (1)	0	33.3% (1)
Endophthalmitis	16.6% (1)	0	33.3% (1)
Hepatic vein thrombophlebitis	33.3% (2)	33.3% (1)	33.3% (1)
Mean duration of antibiotic treatment in weeks (range)	5.16 (3–8)	4.3 (3–6)	6 (4–8)
Mean duration of liver abscess drainage catheter in days (range)	25.27 (9–54)	30.75 (9–54)	18.6 (9–35)
Survival up to hospital discharge	100% (6)	100% (3)	100% (3)

On admission, 50% of patients had abdominal pain, 50% had nausea and vomiting, 33.3% had shortness of breath, 33.3% had altered mental status, 50% had fever, 16.6% had rapid atrial fibrillation, and 33.3% had acute kidney injury. Fifty percent had leukocytosis, 33.3% had severe thrombocytopenia (< 50 K/mcL), and 83.3% had elevated transaminases and alkaline phosphatase (Table 1). All blood cultures were positive for gram‐negative rods on admission day or the following day, and all had speciation and susceptibilities for *K*. *pneumoniae* within 2–3 days.

Eighty three percent of patients initially presented with a single hepatic abscess—four patients with the single abscess in the right lobe and one patient with the single abscess in the left lobe. The remaining single patient had four abscesses in the right lobe. Abscesses were multilocular (50%), heterogeneous (33.3%), and gas‐containing (16.6%). Sizes were between 3.6 and 6.7 cm in diameter. They were identified on admission day (83.3%) and the following day (17%).

Two patients had hepatic vein thrombophlebitis on contrast‐enhanced computed tomography (CT) (Table 1). Both had radiographic improvement with antibiotics and no therapeutic anticoagulation was utilized.

Fifty percent of patients developed distant metastatic infection. Patient A developed emphysematous cystitis, pulmonary septic emboli, complicated parapneumonic pleural effusions, meningitis, ventriculitis, and cerebral septic emboli. Patient B developed urinary tract infection, pulmonary septic emboli, complicated parapneumonic pleural effusion, cerebral septic emboli, and endophthalmitis. A third patient developed pulmonary septic emboli. Patients with metastatic infection were younger (41 vs. 68.6 years), male predominant (100% vs. 0%), and had a higher need for ICU admission and mechanical ventilation (66.6% vs. 0) compared with patients without metastatic infection (Table 1). Pulmonary septic emboli were identified on admission day and the following day, central nervous system (CNS) infections on Days 2 and 5 (two patients) and endophthalmitis on Day 2 (one patient).

The patients requiring ICU admission (Patients A and B) had septic shock, diabetic ketoacidosis (DKA), respiratory failure, severe thrombocytopenia, and altered mental status:

### 3.2. Patient A—Clinical Vignette

A 31‐year‐old male with a history of uncontrolled Type 1 diabetes presented with altered mental status. He was tachycardic, normotensive, and was emergently intubated. Laboratory data were notable for glucose of 491 mg/dL, AST 102 units/L, ALT 128 units/L, total bilirubin 1.4 mg/dL, platelets 12 K/mcL, anion gap 5 mmol/L, lactate 3.2 mmol/L, and pyuria. CT chest/abdomen/pelvis revealed multiple pulmonary cavitary lesions and consolidations suggestive of septic emboli, a 4 × 5 cm gas‐containing liver abscess, gas in the hepatic vein, no biliary pathology, and emphysematous cystitis. Head CT was normal. He was admitted to the ICU on vancomycin and piperacillin‐tazobactam. Blood cultures were positive for *K. pneumoniae*, and liver abscess drainage was deferred due to thrombocytopenia. On Day 2, he developed septic shock. Brain magnetic resonance imaging (MRI) demonstrated multiple areas of sulcal FLAIR hyperintensity and leptomeningeal enhancement suspicious for meningitis, layering fluid with restricted diffusion within the occipital horns of lateral ventricles suggestive of ventriculitis, and multiple cerebral septic emboli (Figure 1). On Day 3, lumbar puncture was performed after platelet transfusion. Cerebrospinal fluid (CSF) studies revealed gram‐negative rods on stain, 444 nucleated cells/mcL with neutrophilic predominance, elevated protein (252 mg/dL), low glucose (43 mg/dL), elevated RBC count (475/mcL), xanthochromia, positive PCR for *K. pneumoniae*, negative culture, and normal intracranial pressure. On Day 5, neurosurgery recommended against external ventricular drain given thrombocytopenia and no hydrocephalus. Repeat CT revealed left pleural effusion, worsening pulmonary septic emboli and hepatic abscess, and right hepatic vein thrombus (Figures 2 and 3). Transthoracic echocardiogram (TTE) showed no endocarditis or atrial thrombus. He underwent liver abscess catheter drainage and bilateral chest tube insertion for complicated parapneumonic effusions. Due to difficulty weaning from the ventilator, a tracheostomy was placed on Day 15. On Day 25, his mentation improved and started to speak. Ophthalmology evaluation on Day 27 revealed no endophthalmitis. On Day 39, the tracheostomy and liver abscess catheter were removed after an abscessogram showed almost complete abscess resolution (Figure 4). The patient was discharged neurologically intact on Day 58, with plans for 8 weeks total of IV ceftriaxone.

**Figure 1:**
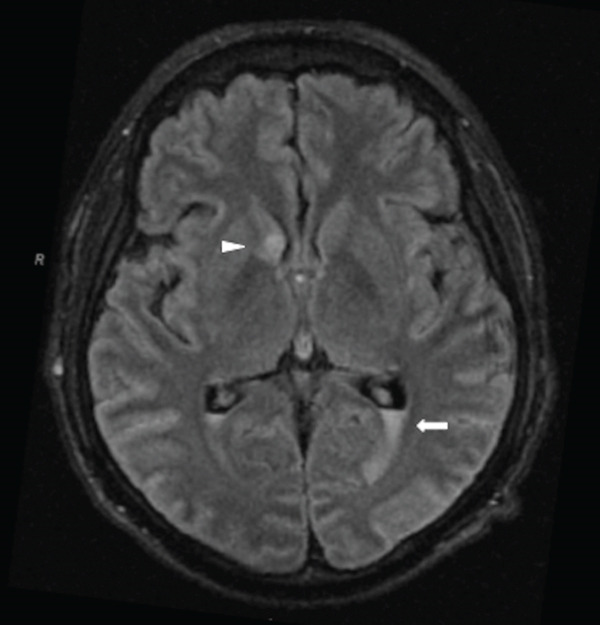
Flair sequence of brain MRI demonstrating moderate layering fluid with restricted diffusion within the occipital horns of the lateral ventricles suggestive of ventriculitis (arrow), and right caudate nucleus septic emboli (arrowhead).

**Figure 2:**
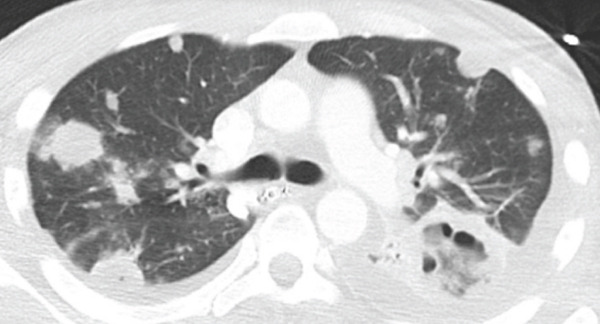
CT scan demonstrating multiple pulmonary septic emboli, one in the left lung with cavitations. Also, there are bilateral pleural effusions.

**Figure 3:**
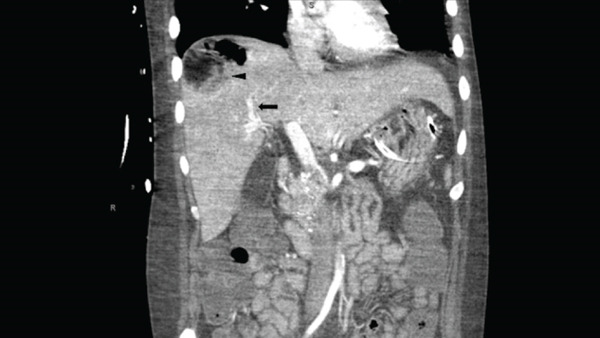
CT scan with contrast demonstrating a complex gas‐containing lateral right hepatic dome abscess measuring 5.1 × 5.9 cm (arrowhead) and a linear hypodensity extending from the right hepatic vein toward this process consistent with septic thrombophlebitis (arrow).

**Figure 4:**
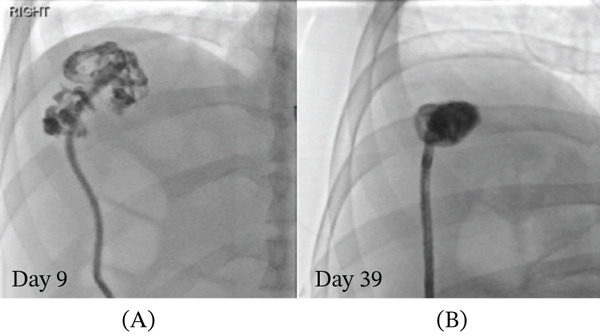
(A) Abscessogram on Day 9 of admission demonstrating a large residual fluid collection and no fistulous communication. (B) Abscessogram on Day 39 after IV antibiotics and catheter exchange demonstrating minimal residual hepatic fluid cavity. Drain was removed after this abscessogram.

### 3.3. Patient B—Clinical Vignette

A 49‐year‐old male with no previous history presented with 6 days of fever, myalgia, and headache. He was somnolent, had rapid atrial fibrillation, and hypotension. Laboratory data were notable for a glucose 450 mg/dL, WBC 7.8 K/mcL with 17% bands, creatinine 2.51 mg/dL, total bilirubin 2.9 mg/dL, AST 99 U/L, ALT 100 U/L, lactate 7.8 mmol/L, beta‐hydroxybutyrate 36.9 mg/dL, and platelets 32 K/mcL. CT chest/abdomen/pelvis revealed diffuse irregular pulmonary nodules consistent with septic emboli, a 4.6 × 2.5 cm hepatic abscess, and no biliary pathology. Head CT was normal. He was admitted to the ICU on IV vancomycin and piperacillin‐tazobactam. Liver abscess drainage was deferred due to solid appearance and thrombocytopenia. On Day 2, blood cultures were positive for *K*. *pneumoniae*, and he developed left eye blurred vision. On ophthalmologic exam, the left eye had hand motion visual acuity, poor pupillary response, full extraocular movements, 2+ conjunctival injection, clear cornea, 2+ cells in anterior chamber, no hypopyon, round and reactive iris, lens with pupillary membrane, very limited view of the fundus, and an attached retina on B‐scan ultrasound. The patient underwent vitreous tap with intravitreal ceftazidime and vancomycin for presumed endogenous endophthalmitis. Vasopressors and insulin drip were weaned off, but on Day 3, he was intubated for lethargy, hypoxemia, rapid atrial fibrillation, and DKA rebound. On Day 5, his mental status improved, and he was extubated. Brain MRI revealed several septic emboli (Figure 5), and transesophageal echocardiogram (TEE) showed no endocarditis or atrial thrombus. Repeat CT on Day 6 revealed a left loculated pleural effusion and liver abscess enlargement. A left‐sided chest tube was placed for a complicated parapneumonic effusion. On Day 12, the patient underwent liver abscess catheter drainage, and 2 days later left‐sided video‐assisted thoracoscopic surgery (VATS) with decortication, with negative pleural cultures. On Day 22, the liver abscess catheter was removed. Repeat ophthalmology evaluation demonstrated a choroid detachment suggesting worsened endophthalmitis and chorioretinitis, but there was no room for further intravitreal antibiotic injections due to the detachment. On Day 30, the patient was discharged with plans for 6 weeks total of IV ceftriaxone. After more than 1 year, his visual acuity remained hand motion only.

**Figure 5:**
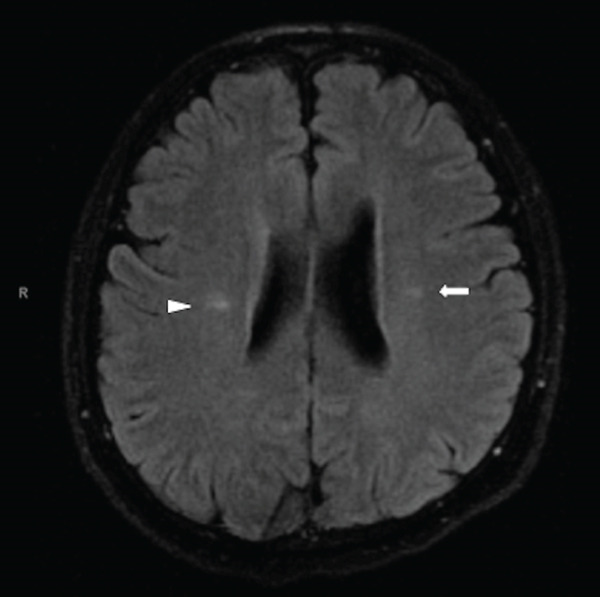
Flair sequence of brain MRI demonstrating cerebral septic emboli (arrow and arrowhead).

Fifty percent of patients initially received ceftriaxone and 50% piperacillin‐tazobactam before switching to ceftriaxone. Thirty‐three percent also received metronidazole. Patients A and B described above also received vancomycin upon ICU admission for broad‐antibiotic coverage in the setting of septic shock until a source of infection was identified. The mean antibiotic course duration was 5.16 weeks (range 3–8 weeks) (Table 1). Two patients received an additional 3 weeks of PO moxifloxacin for endophthalmitis and 2 weeks of inhaled tobramycin for lung involvement, respectively. Abscess cultures of all patients, tracheal aspirate, and urine cultures of the critically ill patients grew *K*. *pneumoniae*. Pleural fluid, vitreal fluid, and CSF cultures were negative. All isolates were pansensitive except to ampicillin. No isolate was extended‐spectrum beta‐lactamase (ESBL) producer.

Each hepatic abscess had percutaneous catheter drainage by an interventional radiologist. Catheter placement was initially deferred in three patients, one due to small abscess size and two due to severe thrombocytopenia. However, each patient developed persistent sepsis and larger abscesses, eventually requiring drainage. One patient developed a fistula from the abscess into the common bile duct that spontaneously healed and an additional abscess that required catheter drainage. Another patient required a catheter exchange after becoming obstructed. All patients had their catheter removed after complete or near‐complete abscess resolution on repeat abscessogram. Mean abscess drainage catheter duration was 25.57 days (9–54 days) (Table 1). All patients had clinical improvement, and there was no in‐hospital mortality. ICU length of stays was between 7 and 19 days.

### 3.4. Secondary Liver Abscess

Three patients from initial data collection had underlying hepatobiliary disease (calculous cholecystitis, acalculous cholecystitis, and choledocholithiasis with cholangitis). They were older than patients with ILAS (76.3 vs. 54.8 years), had no metastatic infection, and one had polymicrobial infection (with *Escherichia coli*). Abscesses were single, multiloculated, and two were pericholecystic. *Klebsiella* isolates were pansensitive, except to ampicillin.

## 4. Discussion

Our patients with ILAS had similar characteristics to patients from Taiwan, South Korea, and the United States, except for a higher metastatic infection rate and lower mortality [1, 4]. Both of our critically ill patients presented with multiorgan metastatic infections (lungs, brain, eye, and urinary tract), DKA, severe thrombocytopenia, altered mental status, septic shock, and respiratory failure requiring mechanical ventilation. Compared to a series of critically ill patients in France [5], our critically ill patients had similar age, male predominance, and high rates of AKI, respiratory failure, and shock. In contrast, our patients had a higher metastatic infection rate (100% vs. 25%) and lower mortality (0% vs. 50%). These differences can be explained by the fact that their case definition was microbiological, and many had pneumonia but not bacteremia or liver abscess. Our patients received similar therapies to those reported in previous studies, including parenteral antibiotics, liver abscess drain placement, and targeted treatment for meningitis, complicated parapneumonic effusions, and endophthalmitis [1].

Although the majority of our patients did not have significant recent travel history, ILAS has been increasingly reported in North America. The source of *K*. *pneumoniae* infection in these patients is hypothesized to be community‐acquired colonization, either via the nasopharynx or gastrointestinal mucosa. In high‐risk patients, bacteria may translocate into the bloodstream and enter the liver through the portal venous system.

With regards to immunosuppression, none of the patients had malignancy, chronic steroid use, chemotherapy, or chronic infection such as HIV. However, 83.3% (5 out of 6) patients had diabetes, which causes immune dysregulation that increases the risk of infection. Of the five patients, three patients had uncontrolled diabetes (defined as A1c > 7*%*). Correlatively, patients who had the most severe uncontrolled diabetes, Patient A (A1c 17.3%) and Patient B (A1c 12.2%), were admitted to the ICU. The third patient who had uncontrolled diabetes had an A1c of 9.4% and distant metastasis as well. Due to medical record limitations or sparse medical care, it was unable to exactly identify how long each patient has had diabetes. All except for one patient, however, had either a new diagnosis of uncontrolled diabetes or at least 7–8 years of diabetes. This suggests that uncontrolled and/or long‐standing diabetes poses a significant risk factor for ILAS infection and severity.

### 4.1. Narrative Review of Critical Care Management Considerations

A high index of suspicion is necessary to identify ILAS early in the disease course. Sepsis presenting with elevated liver enzymes, leukocytosis, and thrombocytopenia should raise the suspicion for ILAS, especially in diabetic patients. The presence of a liver abscess and gram‐negative bacteremia further raises the likelihood of ILAS. It is confirmed when *K*. *pneumoniae* is identified on blood cultures without biliary disease on imaging to suggest a secondary liver abscess.

Diabetes and poor glycemic control are strong risk factors for ILAS and for metastatic infections [8, 9]. It is unclear if strict glycemic control after diagnosis leads to better outcomes. DKA can reoccur if adequate sepsis and source control are not achieved, as seen in one of our patients.

Metastatic infection occurs in up to 28% patients with ILAS [8], commonly in the lungs (16%), eyes (11%), and CNS (8%) [1]. Less commonly, it affects other sites: psoas abscess, necrotizing fasciitis, splenic abscess, prostatitis, osteomyelitis, arthritis, lumbar, or cervical spondylitis and discitis [10–12]. Early identification is crucial for source control and treatment of affected organs, particularly to prevent devastating consequences of ocular and CNS involvement. Only one third of metastatic infections are seen on admission, most within 3 days [13]. A detailed symptomatic review and physical exam are paramount, and focal findings should lead to appropriate clinical and radiological evaluations. Pan‐CT localizes infectious foci in sepsis of unknown origin and can be used in ILAS for metastatic screening or scenarios of persistent sepsis despite treatment [14]. PET scans also localize metastatic infection in ILAS [15]. CT is more sensitive than ultrasound in identifying liver abscesses [16].

### 4.2. Pulmonary Involvement

Septic pulmonary emboli present as diffuse bilateral lesions with various radiographic characteristics: feeding vessel sign (79%), nodules with or without cavities (79%), peripheral wedge‐shaped opacities (64%), patchy ground glass opacities (50%), air bronchograms within a nodule (36%), gas within lesions (36%), consolidations (21%), halo signs (14%), and lung abscesses (14%) [17]. Hemoptysis occurs in up to 14% of patients. Pleural effusions occur in 71% of patients, with 50% requiring tube thoracostomy and 25% requiring VATS and decortication [17]. Lung abscess drainage is rarely needed but has been reported [18].

### 4.3. CNS Involvement

CNS involvement manifests as meningitis, ventriculitis, cerebral abscess, and cerebral septic emboli. Meningitis and brain abscess occur in 4% and 1.7% of patients, respectively [11]. Meningitis presents with fever, altered mental status, nuchal rigidity, and seizures [19]. CSF studies show leukocytosis (mean 1400/uL), hypoglycorrhachia, and hyperproteinorrhachia. Hydrocephalus occurs in 16.6% of patients, and 22% have a concomitant brain abscess [19]. Brain abscesses commonly present with fever, altered mental status, headache, and hemiparesis, and 60% have meningitis. Most abscesses are supratentorial, single, and 10% contain gas. The most common CT pattern is ring‐enhancing lesions surrounded by edema. Most patients are treated with brain abscess aspiration [11, 20]. Ventriculitis is less common. It is usually identified on CT or MRI as dependent hyperdense layering material in the occipital horns of the lateral ventricles in patients with meningitis [21]. Cerebral septic emboli are usually seen on brain MRI but not CT. Clinical correlation is important to identify embolic lesions as septic emboli instead of embolic strokes, as therapeutic anticoagulation can lead to hemorrhagic transformation, as similarly seen in endocarditis [22]. Cervical and lumbar spondylitis causing quadriparesis and paraparesis have been reported [11]. In patients with ILAS with altered mental status, seizures, nuchal rigidity, or focal neurological deficits, contrast‐enhanced head CT should be obtained, followed by lumbar puncture if no contraindications. Contrast‐enhanced brain MRI is required for suspicion of brain abscess, cerebral septic emboli, or ventriculitis, as visualization on head CT is not guaranteed. Neurosurgical consultation is warranted for brain abscesses, hydrocephalus, or ventriculitis for consideration of surgical drainage, abscess aspiration, or external ventricular drainage. There is limited data on adjunctive intrathecal or intraventricular antibiotics in ILAS ventriculitis, but they are not routinely recommended in nosocomial ventriculitis, except in refractory or resistant infections [23].

### 4.4. Ocular Involvement

*Klebsiella pneumoniae* endogenous endophthalmitis (KPEE) is the most common cause of endogenous endophthalmitis in Asian countries (58% of cases), but it is an uncommon cause in Western countries (4.3%) [24]. Endogenous endophthalmitis in ILAS (7.3%–11%) is more common than in MRSA bacteremia (0.2%) and candidemia (1.8%) [1, 24–26]. Symptoms are blurry vision (47%), eye redness (31%), pain (10%), and floaters (5%), and 36% of patients have no ocular symptoms [27, 28]. Mean time from sepsis onset to ocular symptoms is 4.4 (+/−3.0) days [29]. On exam, 79% have conjunctival injection, 37% had hypopyon, and 74% had loss of fundal view. Most patients have severely impaired visual acuity, commonly hand motion only, and 26% of cases are bilateral [28]. Other exam findings are corneal edema, anterior chamber cells, iritis, and vitritis. The treatment is intravitreal antibiotics in addition to intravenous antibiotics, which should start as soon as endophthalmitis is suspected. Cultures should be obtained during vitreous tap, although the yield is low, 37.5% [28]. Repeat intravitreal antibiotics injections can be considered after 48–72 h in severe cases [29]. Early vitrectomy has been suggested as it may improve outcomes, but it is usually only recommended in severe cases [30]. The prognosis of KPEE is poor. In a KPEE series, 80.2% had poor vision (< 4/200), and 26.8% required evisceration [29]. Hypopyon and treatment delay are associated with worse prognosis [31]. Due to its high prevalence, high rate of asymptomatic patients, and as early diagnosis and therapy improve visual outcomes [32], different authors have recommended endophthalmitis screening for patients with ILAS [27, 28, 33].

### 4.5. Liver Abscess

Compared with pyogenic liver abscesses, ILAS liver abscesses are more frequently characterized as single, monomicrobial, multilocular, solid‐appearing, involving a single hepatic lobe, associated with thrombophlebitis, presenting gas in the abscess, and having less pus on needle aspiration [34–36]. Image‐guided percutaneous catheter drainage is recommended compared with aspiration alone, as it decreases time to recovery [37]. Specimens should be sent for aerobic and anaerobic cultures. ILAS abscesses may not initially be amenable to drainage as they often appear solid. However, delaying abscess drainage can lead to persistent sepsis, which occurred in three of our patients. We recommend inserting a catheter upfront, regardless of consistency, as eventually the abscess will liquefy. The catheter should be flushed periodically as it can become obstructed [38]. Complete or near‐complete drainage demonstrated by follow‐up abscessogram should be obtained before catheter removal, as abscesses can recur. Surgical drainage and hepatic resection have been performed after percutaneous drainage failure with need for source control [39, 40]. Septic thrombophlebitis occurs in 30% of ILAS patients, in the hepatic veins (95%) and in the portal vein (5%), known as pylephlebitis. Recanalization or improvement occurs in 73% of cases after antibiotic therapy, including pylephlebitis, and thus anticoagulation is not recommended [35, 41].

### 4.6. Antibiotic Treatment

*Klebsiella* isolates in ILAS are generally sensitive to cephalosporins [42]. Resistant isolates, including ESBL‐producing, and carbapenem‐resistant strains, are uncommon, but frequency has increased [43–45]. Initial antibiotic regimen before microbiological confirmation should include gram‐negative and anaerobic coverage as follows: for pyogenic liver abscess (i.e., ceftriaxone + metronidazole, piperacillin‐tazobactam, fluoroquinolone + metronidazole, or carbapenem). Following organism identification and antibiotic susceptibility testing, therapy can be tailored, usually to a third‐generation cephalosporin, ampicillin–sulbactam, or a quinolone [1]. A third‐generation cephalosporin is preferable to a first‐generation cephalosporin, as its use is associated with lower rates of metastatic infection, complications, and mortality [46]. Recommended treatment duration is usually 4–6 weeks. Parenteral administration is recommended for 2–3 weeks and until clinical improvement and complete or near‐complete liver abscess resolution, followed by oral for the remainder of the treatment. However, patients with CNS, ocular, or other severe organ involvement usually need longer parenteral antibiotic courses, with individualized duration depending on clinical response [1, 19, 20]. A randomized controlled trial in ILAS without uncontrolled sepsis, CNS, or ocular involvement found no difference in outcomes between oral ciprofloxacin versus intravenous ceftriaxone for 28 days following 5 days of parenteral antibiotics [47]. In CNS infection, third‐generation cephalosporins are the drug of choice due to superior CSF penetration. A dose of 2 g IV BID of ceftriaxone is recommended. Imipenem and meropenem can be used for ESBL‐producing strains in patients with CNS infections [1]. Endophthalmitis requires additional intravitreal antibiotics. Systemic antibiotics that achieve therapeutic levels in the vitreous include meropenem, moxifloxacin, and ceftriaxone [48]. Third‐generation cephalosporins were the most commonly used antibiotics in a KPEE series [49]. Hospital‐acquired infections with hypervirulent *K*. *pneumoniae* (usually ventilator‐associated pneumonia) have been reported in China. They are usually multidrug‐resistant, including ESBL‐producing, carbapenem‐, and colistin‐resistant strains [50–52].

### 4.7. Prognosis

ILAS′ prognosis is overall favorable but metastatic infections, particularly CNS and endophthalmitis, carry significant morbidity. Mortality rates in Taiwan, South Korea, and the United States are between 4% and 8% [1]. The mortality of ILAS with pulmonary infection is 14% [17] and with CNS or ocular infection is 9.1% [53]. Mortality risk factors are metastatic infection, APACHE II⩾16, septic shock, acute respiratory failure, gas on imaging, and resistant strains [13, 54]. Patients with CNS or ocular involvement have high rates of disability, as demonstrated in a patient series in which 70% had impaired higher cortical function, quadriplegia, paraparesis, or loss of vision [5]. Despite this, a nonnihilistic approach should be taken in the care of patients with ILAS to avoid falling into a self‐fulfilling prophecy of poor prognosis. In our experience, a high level of support and expectant management is warranted. For example, our patient with meningitis and ventriculitis took 25 days to regain consciousness and had full neurologic recovery.

## 5. Limitations

The small number of cases is a limitation of this study. With six patient cases identified, 83.3% had diabetes. Of the three patients who had metastatic distant infection, all three had pulmonary septic emboli, two had parapneumonic effusion, UTI, and cerebral septic emboli, and one had ocular involvement. As a result, it is challenging to extrapolate if metastasis to specific organs could guide clinicians in imaging and laboratory workup. Similarly, variation existed in patient symptom presentation. In the three patients who had local liver abscesses, prevalent symptoms included abdominal pain, nausea, vomiting, fever, and leukocytosis, in comparison with their less symptomatic counterparts who had distant metastatic infection. One possible explanation is that localized infection triggers a more sustained and concentrated immune response; meanwhile, systemic infection mounts a less concentrated response. This is especially supported by the immune suppression seen in the patients with uncontrolled diabetes who were found to have systemic metastasis.

It was noted that patients who had distant infection had a shorter mean days of drainage catheter in place (18.6 days) when compared with localized hepatic abscess (30.75 days). One outlier in the localized infection group required a second drain placement due to persistent abscess, which was ultimately in place for 54 days. The abscess size and characteristics did not appear to be significant factors in drain duration.

Another limitation of this study was the lack of microbiological testing for the hypervirulent serotypes (such as K1 and K2) given the community‐based hospital setting in which these cases are described. However, given the documented evidence that hypervirulent strains are more likely to cause invasive and disseminated infection in the pattern of liver, CNS, pulmonary, and ocular systems, it can be reasonably inferred that these strains may have been present [4]. Despite these limitations, these patient presentations provide a closer understanding of how ILAS presents and its clinical course in Northern America.

## 6. Conclusion

In conclusion, ILAS is an emerging disease that causes severe critical illness with multiorgan involvement and requires a high index of clinical suspicion. Pan‐CT can identify metastatic infection. Neurologic symptoms and signs should prompt head CT, lumbar puncture, and brain MRI. Endophthalmitis screening is recommended in all patients. Treatment includes prolonged antibiotic therapy, liver abscess catheter drainage, source control of all involved organs, and early consultation with ophthalmology, infectious disease, neurology, and neurosurgery as indicated. ILAS prognosis is good overall, but with the caveat that ocular or CNS involvement can lead to significant residual disability.

## Funding

No funding was received for this manuscript.

## Disclosure

This paper was independently performed without outside influence and was purely conducted through available published literature.

## Consent

No written consent has been obtained from the patients as there is no patient identifiable data included in this case series.

## Conflicts of Interest

The authors declare no conflicts of interest.

## Data Availability

The data that support the findings of this study are available from the corresponding author upon reasonable request.
